# Clinical and Pathological Features and Survival Outcomes of Breast Cancers with Intermediate ER Expression

**DOI:** 10.3390/cancers17132252

**Published:** 2025-07-05

**Authors:** Jonathan Hammond, Nicholas Lambert, Ioannis A. Voutsadakis

**Affiliations:** 1 Northern Ontario School of Medicine, Sudbury, ON P3E 2C6, Canada; jonhammond@nosm.ca (J.H.); nlambert@nosm.ca (N.L.); 2 Algoma District Cancer Program, Sault Area Hospital, Sault Ste. Marie, ON P6B 0A8, Canada; 3 Section of Internal Medicine, Division of Clinical Sciences, Northern Ontario School of Medicine, Sudbury, ON P3E 2C6, Canada; 4 Holden Comprehensive Cancer Center, University of Iowa Hospitals and Clinics, 200 Hawkins Dr., Iowa City, IA 52240, USA

**Keywords:** breast cancer, ER expression, ER-intermediate, ER expression level, prognosis

## Abstract

Background: Breast cancer is the most prevalent women’s cancer, representing about a third of all cancers diagnosed in women. Estrogen receptor (ER) is an important transcription factor expressed in 70% to 75% of all breast cancers. Of these breast cancers, the majority express ER robustly in 91–100% of tumor cells. However, a minority of breast cancers express ER in intermediate levels between 11% and 90% of tumor cells. The characteristics and outcomes of this intermediate subset of ER-positive breast cancers are not well studied. Methods: We retrospectively reviewed the medical records of all breast cancer patients treated in our Cancer Center over a period of 8 years. Patients were grouped according to the level of ER expression in tumors with negative/low ER expression, intermediate ER expression, and high ER expression, and the groups were compared for tumor characteristics and outcomes. Results: Patients with high ER levels (91% to 100%) represented 75.6% (600 of 794 patients), patients with intermediate ER expression represented 11.5% of the entire group (91 of 794 patients), and patients with negative/low ER expression (ER expression 0–10%) represented 12.9% of the entire cohort (103 of 794 patients). Patients with intermediate ER expression presented at a younger age than patients with high-ER cancers, as well as a more advanced stage and higher grade. These characteristics were more akin to patients with negative/low ER levels. Mastectomy was the surgical method of resection more commonly in ER-intermediate tumors than in ER-high tumors. The relapse-free survival (RFS) of patients with ER-intermediate cancers was worse than the RFS of patients that expressed ER robustly. Conclusion: The level of expression of ER defines groups of patients with varying characteristics and prognoses. Patients in the intermediate ER expression group had notable differences from patients in the high ER expression group including younger age, (more often) a higher grade, and low PR positivity. Differences observed between the group of patients with intermediate ER expression and that with high ER expression may help to prioritize therapeutic algorithms.

## Simple Summary

Most breast cancers express the estrogen receptor (ER), and among the ER-positive cancers, the majority express the receptor in almost all (91% to 100%) tumor cells. The smaller groups of patients that express a lower percentage of ER are divided into a group with low ER expression (1% to 10%) and an intermediate ER group (ER expression of 11% to 90%). The low-ER group is better characterized, and guidelines recommend that low ER expression should be explicitly mentioned in pathological reports. The intermediate ER group is less extensively studied. This group is the subject of this study, with the aim of reporting particular characteristics and outcomes that set it apart from the high ER expression group.

## 1. Introduction

Breast cancer is one of the most prevalent cancers, and although it has a better prognosis than other malignancies, it is a significant cause of morbidity, mortality, and public concern [1]. Classification of breast cancers according to the expression of the receptors ER (estrogen receptor) and HER2 (human epidermal growth factor receptor 2) has helped to describe clinically relevant sub-types of breast cancers and has been instrumental in devising successful targeted therapies [2]. The most prevalent sub-type of breast cancers is positive for ER and negative for the HER2 receptor, representing three-quarters of all breast cancers. Most of these ER-positive/HER2-negative breast cancers have a more indolent course than breast cancers that are HER2-positive or triple-negative (not expressing ER, the progesterone receptor (PR), or HER2) [3]. The clinically defined breast cancers based on the expression of ER, PR, and HER2 overlap to a significant degree with the genomic classification of breast cancers, based on gene expressions, which has classified breast cancers into four categories: luminal A, luminal B, HER2-enriched, and basal-like [4,5]. In the genomic classification, the two luminal sub-types encompass most ER-positive breast cancers but differ in other clinical and pathologic characteristics, such as prevalence of PR and HER2 positivity and grade [4,6]. Luminal B cancers tend to have lower PR expression, include many ER-positive cancers with HER2 positivity, and be of higher grade. Moreover, they express higher levels of the proliferation marker Ki67. In contrast, luminal A cancers are lower-grade and usually express higher levels of PR. In addition, luminal A cancers display a higher sensitivity to anti-estrogen hormonal therapies, such as tamoxifen and aromatase inhibitors [7]. Breast cancers that are HER2-enriched include most of the cancers that are HER2-positive/ER-negative and several cancers that are HER2-positive and also ER-positive. The genomic basal-like category overlaps with the clinical triple-negative phenotype [8].

Alongside differences in PR expression, ER-positive luminal cancers differ in the level and intensity of ER expression [9]. Luminal A cancers mostly display a strong, homogeneous expression of ER in all tumor cells on histologic sections, while some luminal B cancers have a more heterogeneous and lower intensity of ER expression. Despite these differences, the clinical and therapeutic implications of a lower expression of ER have not been examined in detail, except for cancers with expression of ER in the lower spectrum (1–10%). These low-ER-expressing cancers have been confirmed to behave more similar to ER-negative cancers and segregate genomically with basal-like cancers [10]. However, breast cancers with intermediate expression of ER (11–90%) have attracted less interest, and their natural history and differences from cancers with high ER expression (91–100%) are less often discussed. In this article, we will examine breast cancers with intermediate expression of ER (11–90%) with the goal of identifying differences that set them apart from high ER expressors and may have potential therapeutic repercussions.

## 2. Patients and Methods

We undertook a retrospective analysis of all breast cancer patients treated in our Cancer Program between 2016 and 2024. Inclusion criteria for this study included a histologic diagnosis of breast cancer and availability of ER percentage and ER intensity evaluation, assayed by immunohistochemistry in the initial diagnostic biopsy. Exclusion criteria consisted of lack of availability of detailed ER expression data, diagnosis with bilateral cancers, and incomplete follow-up after diagnosis. Follow-up was considered complete if a patient was followed until death or was seen within the last year from data collection. A total of 794 patients met the criteria for the study, and their medical records were reviewed. The following data were collected: patient demographics, tumor characteristics (stage, grade, and ER, PR, and HER2 status), treatments (surgery, radiation therapy, and systemic treatments) and survival status. For some parameters, data were missing in patients’ records, and therefore evaluations and comparisons included a smaller number of observations. Detailed ER, PR, and HER2 receptor expression information was extracted from the pathology reports. The methods employed for the evaluation of the three receptors remained unchanged during the time period of the study. HER2 status was considered negative if the score was 0, 1+ or 2+ in immunohistochemistry (IHC), with negative amplification by fluorescence in situ hybridization (FISH) in the case of 2+ by IHC. HER2 was considered positive if 2+ by IHC with positive FISH or if 3+ by IHC, according to the American Society of Clinical Oncology/College of American Pathologists (ASCO/CAP) guidelines [11]. HER2 IHC 3+ positivity required strong circumferential membranous staining in more than 10% of tumor cells [12]. HER2 FISH positivity was defined as a HER2 to CEP17 (chromosome 17 centromeric probe) ratio of 2 or above or a mean HER2 signal of 6 and above per nucleus. Reports on the genomic Oncotype Dx test were recorded when available. Oncotype Dx was performed in patients with T1 and T2, either lymph node-negative or lymph node-positive, with up to 3 positive lymph nodes ER-positive/HER2-negative cancers, according to the standard clinical indications of the test [13].

Patients were categorized into three groups according to the tumor ER percentage expression. The ER-high group was defined as tumors expressing ER in 91–100% of tumor cells, the ER-intermediate group was defined as expressing ER in 11–90% of tumor cells, and the ER-low/negative group as not expressing ER or expressing it in 1–10% of tumor cells. The rationale of defining the intermediate group as consisting of cancers with ER expressions between 11% and 90% was based on the fact that cancers with lower ER expression (1–10%) are comparatively well established in the literature as constituting a separate group and are required by ASCO/CAP guidelines to be explicitly reported as low-positive, while the dominant group with the strongest ER expression were documented as 91% to 100% in pathologic records in our series [14]. Evaluations were performed in a semi-automated manner by experienced pathologists with expertise in breast cancer in a CLIA (Clinical Laboratory Improvement Amendments)-certified clinical pathology laboratory. Periodic internal audits of selected cases were performed as per the pathology laboratory quality control protocols. Hormonal therapies in the adjuvant setting were given for 5 to 10 years according to guideline recommendations.

Characteristics of the patients, the tumors, and the outcomes of the three studied groups were compared. The ER and PR histoscore was calculated by multiplying the percentage of hormone positive cells in immunohistochemistry sections by 3 for a strong intensity of staining, by 2 for a moderate intensity of staining, and by 1 for a weak intensity of staining. Therefore, the histoscores were calculated by taking into account both the percentage of positive cells and the intensity of staining and ranged from 0 in ER or PR-negative cases to 300 in cases with a strong expression of the receptors in 100% of tumor cells.

The primary survival outcomes of interest were recurrence-free survival (RFS) and overall survival (OS). RFS was defined as the time interval in months from the date of diagnosis to the date of recurrence or death, whichever occurred first, or censored at the patient’s last follow-up without recurrence. OS was defined as the time interval in months from the date of diagnosis to death or censored at the last follow-up of the patient. Survival plots were constructed using the Kaplan–Meier method and compared using the log rank test. A Cox regression proportional hazard multivariate analysis was performed to identify statistically significant factors associated with overall survival and recurrence-free survival. In these analyses, ER and PR variables were entered as percentage of positive tumor cells. Descriptive statistics, including means, medians, confidence intervals, and interquartile ranges, were used for the summary of the variables of interest. For comparing variables of interest, continuous parameters were compared using an ANOVA or the Kruskal–Wallis test, and categorical variables were compared using the χ^2^ test or Fisher’s exact test. All resulting *p* values were considered significant at the level of *p* < 0.05.

The study protocol obtained regulatory approval from the research ethics board of the institution.

## 3. Results

**Figure 1:**
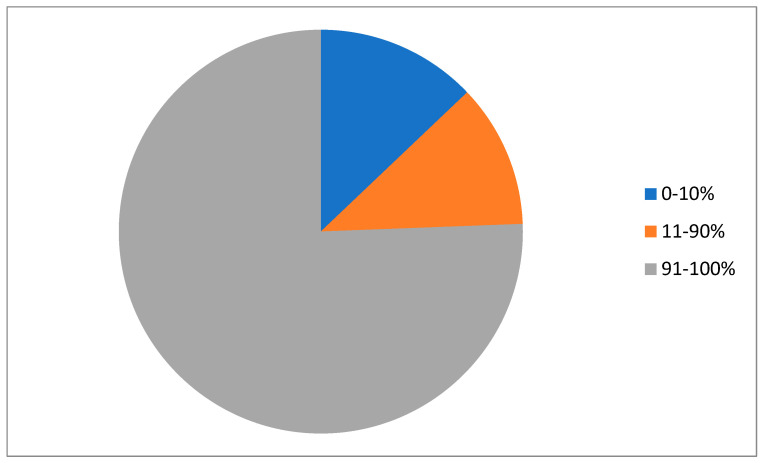
Schematic representation of the percentage of patients with each ER expression category. In blue are the 12.9% of patients in the series who had negative/low ER expression (0 to 10% of tumor cells) cancers. Some 11.5% of patients had intermediate ER expression (11% to 90% of tumor cells, orange), and 75.6% of patients had cancers with high ER expression (91% to 100% of tumor cells, gray).

A total of 794 patients with breast cancer diagnosed and treated in our institution met the inclusion criteria and were therefore included in the data analysis. Patients were categorized into three groups according to the level of ER expression in the tumors. The ER-negative/low group (ER expression 0–10%) included 103 patients (12.9%, Figure 1); 91 patients (11.5%) were in the intermediate-ER group (ER expression 11–90%); and 600 patients (75.6%) belonged to the high-ER group (ER expression 91–100%). The mean age of all the patients was 65.1 years, with a significant difference (*p* < 0.00001) between the groups (Table 1). ER-negative/low and ER-intermediate breast cancers were diagnosed at a younger age (with mean ages of 61.6 and 59.8 years old) compared to the mean age of the high-ER group, which was 66.5 years old. Regarding menopause status, there was a statistically significant difference between groups (*p* < 0.00001). Patients with intermediate-ER breast cancers were diagnosed after menopause in 75% of cases, while patients in the high-ER group were post-menopausal at diagnosis in 91% of cases. There was also a statistically significant difference (*p* < 0.00001) in the method of detection between the three groups (Table 1). In addition, a statistically significant difference in the rates of hypertension was observed (*p* = 0.02), with highly ER-positive breast cancer patients having a 10% higher rate of hypertension than the patients in the other two groups; this is possibly related to the more advanced age in the former group.

Regarding tumor characteristics, ER-negative/low tumors and ER-intermediate tumors were less frequently stage I and more frequently stage IV (*p* = 0.00001, Table 2). Stage IV cancers were observed in 26% of the ER-negative/low group, in 25% of cases in the ER-intermediate group, and in 6.6% of cases in the ER-high group (*p* < 0.00001). In terms of grade, there were also statistically significant differences between the groups. Out of the 103 ER-negative/low tumors, none were grade 1, whereas 76 (89.41%) of ER-negative/low tumors were grade 3. In the ER-intermediate group, about two-thirds of patients (62.5%) had tumors of a high grade. This contrasted with the ER-high breast cancer group, in which over three-quarters of the tumors were grades 1 and 2, but only 22.6% were grade 3 (Table 2). There was no statistically significant difference in perineural infiltration, but there were statistically significant differences for lymphovascular invasion, with the ER-intermediate group having the highest prevalence (*p* = 0.01). The PR expression was also lower in the ER-intermediate group compared with ER-high tumors (*p* < 0.00001). A PR histoscore above 60 was not observed in any patients with ER-negative/low expression and was present in 33% of patients in the ER-intermediate group and 73.5% of patients in the ER-high expression group (Table 2). HER2 positivity showed statistically significant differences between the groups (*p* < 0.00001). The rate of HER2-positive tumors was 6.5% for ER-high breast cancers, whereas the rates were 24% and 36% for ER-negative/low and ER-intermediate tumors, respectively. The Oncotype Dx test was performed in 284 patients in the series. All four patients in the ER-negative/low group with an Oncotype Dx test performed had a high recurrence score (RS > 24). In the ER-intermediate group, over half of the patients (52.4%) had a high RS, while only 9.3% of the patients in the ER-high group had a high RS (*p* < 0.00001, Table 2).

Within the ER-intermediate group, most patients (61 of 91, 67%) had ER expression in the higher range (61% to 90%), while 12.1% (11 of 91 patients) had ER expression between 31% and 60%, and 20.9% (19 of 91 patients) had ER expression between 11% and 30% (Supplemental Table S1). Although the numbers in each group were small, thus decreasing our confidence in the comparisons, significant differences between the three intermediate-range groups were observed in the prevalence of higher PR histoscores (more prevalent in the high/intermediate group) and in HER2 positivity (more prevalent in the two higher intermediate-ER groups, as shown in Supplemental Table S2).

Regarding therapies received by the patients in the three groups, there were statistically significant differences between the types of surgery (lumpectomy or mastectomy, *p* < 0.00001, Table 3). ER-negative/low breast cancer patients had the lowest rates of lumpectomy (46.15%) followed by the ER-intermediate group (62%) and the ER-high group (75%). The rates were reversed for mastectomy, with the ER-high group having the lowest mastectomy rates (24%) followed by the ER-intermediate (37%) and the negative/low group (52%). For adjuvant therapies, there were statistically significant differences in the rates of adjuvant hormonal therapy (*p* < 0.00001), adjuvant radiation (*p* = 0.02), and adjuvant chemotherapy (*p* < 0.00001). Intermediate-ER breast cancers had lower rates of both adjuvant hormonal therapy (80.5%) and radiotherapy (64.4%) when compared to high-ER cancers but higher rates of chemotherapy (52% versus 24.7% in cancers with high ER) (Table 3).

With a median follow-up for surviving patients of 56.5 months, there were statistically significant differences in OS and RFS rates between the three groups with varying ER expression. The mortality rates were 29.1% and 23.1% for ER-negative/low and ER-intermediate tumors, respectively, and 12% for ER-high tumors (*p* < 0.00001) (Table 4). The recurrence rates were also statistically significantly different between the groups (*p* < 0.00001), with the highest rates of recurrence in ER-negative/low tumors (28.9%) followed by the ER-intermediate group (16.5%) and the ER-high group (8.4%). The median time to recurrence was statistically significant different between the groups (Kruskal–Wallis test, *p* = 0.001). The median time to recurrence was about 38 months in the ER-negative/low group and 32 months in the ER-intermediate groups compared to 49 months in the ER-high tumors. The median survival time was also longer in the group with high ER expression (Table 4).

**Figure 2:**
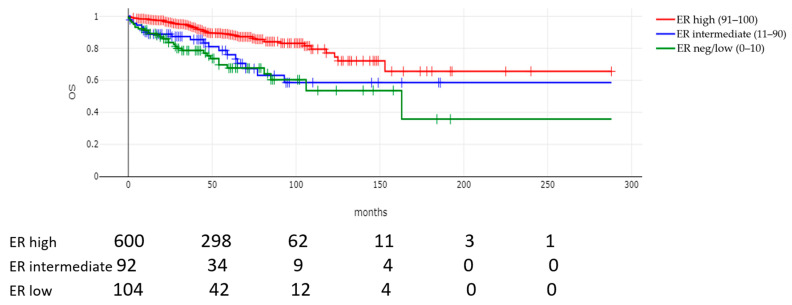
Overall survival (OS) according to level of ER expression. Log rank *p* = 0.00002. Numbers at risk are provided below the curves. The median survival time was 162.3 months in the ER-negative/low group (green line) and was not reached in the ER-intermediate group (blue line) and the ER-high group (red line).

**Figure 3:**
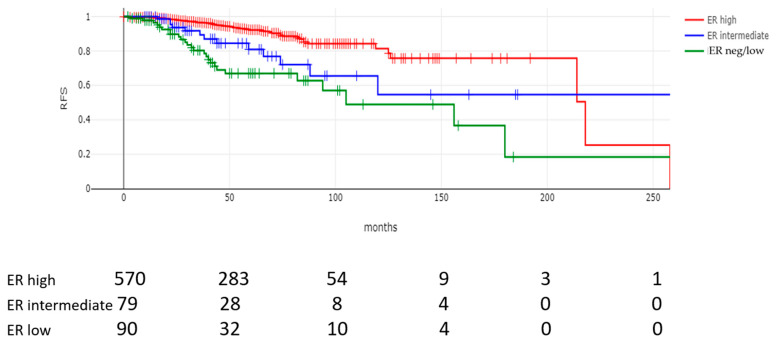
Relapse-free survival (RFS) of stage 1 to 3 (stage 4 patients were excluded) breast cancer patients according to level of ER expression. Log rank *p* < 0.0001. Numbers at risk are provided below the curves. The median time to relapse for relapsing patients was 102 months in the ER-negative/low group (green line) and was not reached in the ER-intermediate group (blue line) and in the ER-high group (red line).

These results were also substantiated with the construction and comparison of Kaplan–Meier curves. The OS of patients with ER-negative/low levels and with intermediate ER levels in their tumors was worse than the OS of patients with ER-high tumors (log rank *p* < 0.0001, Figure 2). Similarly, the RFS of patients with stage 1 to 3 breast cancers and negative/low or intermediate ER expression was worse than the RFS of patients with high ER expression (log rank *p* < 0.0001, Figure 3). Multivariate Cox regression analysis for OS confirmed the independent value of ER level as a continuous variable—alongside tumor stage and grade and patient age—in a model that included age, ER percentage expression, stage and grade as variables (Supplemental Table S3). However, in a more extensive multivariate model that included (in addition to the four variables) PR expression, histology, and hormonal therapy administration, ER expression lost statistical significance, while age, stage, and grade remained significant (Table 5). In addition, hormonal treatment was associated with a significant reduction in the risk of death (hazard ratio: 0.40, 95% CI: 0.19–0.84, *p* = 0.01), suggesting that a co-linearity of ER expression and hormonal therapy administration may exist and that hormonal therapy may decrease the effect of ER positivity on survival. In a multivariate Cox regression model for RFS, with age, ER percentage expression, stage, and grade as variables, stage and ER expression levels retained significance (*p* = 0.0001 for both), while tumor grade and patient age were not significant (Supplemental Table S4). ER expression, together with stage, was also significantly associated with RFS in a more extensive multivariate Cox regression model, which also included PR expression, histology, and hormonal therapy administration (Table 6), suggesting that, on average, every 10% increase in ER expression decreased the relapse probability by 3%.

## 4. Discussion

ER is a transcription factor of the nuclear receptor super-family and belongs—together with the PR, the androgen receptor (AR), the glucocorticoid receptor (GR) and the mineralocorticoid receptor (MR)—to the steroid receptor sub-family [15]. These receptors remain in the cytoplasm in an inactive state if not coupled with the respective steroid hormone ligands. When bound by their ligands, nuclear receptors change their three-dimensional conformation, uncovering a nuclear localization signal, and enter the cell nucleus where they bind specific sequences of DNA in target gene promoters as homodimers and promote transcription [16]. For ER, the target DNA-binding sequence consists of the oligonucleotide 5′-GGTCAnnnTGACC-3′, where n is any nucleotide.

Most breast cancers are ER-positive and are treated with hormonal therapies alone or in combination with other targeted drugs [17]. The majority of metastatic ER-positive breast cancer patients bear tumors that express the receptor robustly and respond well to combinations of hormonal therapies with CDK4/6 inhibitors or other targeted therapies; they often derive significant clinical benefit from these therapies [18,19]. In contrast, the smaller group of ER-positive patients with a low ER expression (in ≤10% of tumor cells) benefit less from hormonal-based therapies and have outcomes more similar to the aggressive triple-negative breast cancer type [10]. The behavior of ER-positive patients with intermediate expression of ER (11% to 90%) is less predictable, and these patients have less often been the subject of systematic studies.

In the current study, we reviewed all the patients with intermediate ER expression diagnosed and followed in our center in the most recent 8-year period and compared them with patients who have breast cancers higher and lower on the ER expression spectrum. Patients with intermediate ER expression were younger and less likely to have stage I tumors but were more likely to have metastatic tumors compared to patients expressing ER more robustly. Higher grade and lower PR expression were also more common with ER-intermediate tumors. These high-risk features are associated with worse survival outcomes. Our findings agree with recent series that have reported lower PR positivity and a higher prevalence of grade 3 cancers when the ER positivity is intermediate or low [20,21]. From a genomic perspective, ER-positive breast cancers with intermediate levels of ER expression (between 11% and 90%) have been shown to form a distinct group belonging more frequently to the luminal B genomic profile as well as to the two non-luminal types (HER2 enriched and basal-like) [22,23]. In contrast, the more prevalent group that expresses the ER receptor more robustly (90–100%) aligns most frequently with the genomic luminal A profile, while the ER-low group that expresses the ER in 1% to 10% of cells aligns with the basal-like genomic subgroup [24].

Several therapeutic implications pertinent to patients with intermediate ER expression can be deduced from these data. First, the effectiveness of hormonal therapies may be inferior in a subset of patients with intermediate ER expression, especially in the lower range of the spectrum [25]. This has been confirmed for ER-low breast cancers with expressions between 1% and 10%, which behave similar to triple-negative breast cancers, despite being conventionally treated with hormonal agents [10]. In ER-positive post-menopausal patients treated with adjuvant tamoxifen as monotherapy and stratified according to the ER histoscore, the 10-year OS of patients with a histoscore below 50 was 41%. For those with a histoscore between 50 and 100, the OS was 71%; for those with a histoscore between 100 and 200, the OS was 67%; and for those with a histoscore above 200, the 10-year OS was 84% [26]. In a study that examined 2 years versus 5 years of adjuvant tamoxifen and showed that long-term mortality was reduced with longer tamoxifen treatment, the benefit was derived by patients with lower (below median) ER levels [27]. By contrast, patients with ER expression above the median had similar survival outcomes with 2 years and 5 years of adjuvant tamoxifen. This observation implies that due to lower sensitivity to endocrine treatment, breast cancers with lower ER levels may require longer exposure in the adjuvant setting to derive the same benefit. However, the method used in this study for ER measurement was an older enzyme immunoassay that is no longer in routine clinical use; therefore, the corresponding cut-offs for extrapolation to the IHC methods in current use cannot be easily ascertained [28]. Consistent with a lower sensitivity to ER therapies, ER-intermediate cancers have a higher prevalence of Oncotype Dx high recurrence score (RS). About half of the patients with ER-intermediate breast cancers had an Oncotype RS above 24, while only about 10% of patients with high ER expression had an RS above 24. A second therapeutic consideration derived from the pathologic characteristics of ER-intermediate cancers is the rate of HER2 positivity, which is higher, suggesting that a subset of these more aggressive tumors would benefit from addition of anti-HER2 targeting treatments, which are clinically available and effective [29,30,31].

There are some limitations to our study. The evaluations of the cohort were performed retrospectively, and the potential for bias, including selection bias, is associated with this design. Selection-derived bias was partially addressed by the fact that all patients with data available in the period of the study were included in the analysis as per the study protocol. For some parameters, a significant number of patients had no data in the retrospective database, thus introducing a potential source of selection bias and reduced statistical power. Moreover, the study included a single-center cohort of patients, reflecting local practice patterns and treatment algorithms, which may restrict the generalizability of findings to other cohorts. Local practices may differ regarding the degree of reliance on clinical and pathologic characteristics or on genomic assays for therapeutic decisions; they may also differ in their choices of first-line treatments for patients who are expected to be less sensitive to hormonal therapies. Reassuringly, though, available data from other ER-intermediate cohorts concur with several of the findings in our cohort. Although a large number of patients were examined in the current study, the cohort with intermediate ER expression was smaller, as only 11.5% of breast cancer patients had tumors with intermediate ER expression. However, this still represents one of the largest cohorts of ER-intermediate breast cancer patients reported in the literature. The specific rationale for the choice of treatments was not consistently available in the patients’ records and could not always be ascertained. Therefore, it remains unclear if the level of ER expression specifically influenced patient treatment strategies. Moreover, the duration and specific types of hormonal treatments were not available in the study database for analysis. Analysis of Ki67, a proliferation marker commonly used to distinguish between luminal A and luminal B cancers, was also not performed during the period of the study and was not available for analysis. However, Ki67 is part of the gene panel used to derive the Oncotype Dx score and was therefore partially captured in cases that had the Oncotype Dx test available.

## 5. Conclusions

In conclusion, breast cancer patients with intermediate ER expression have characteristics and outcomes that require specific considerations in their management, with particular attention to the possibility that many of these patients may display an inferior sensitivity to hormonal therapies. Other targeted therapies could be valid alternatives for the treatment of these patients.

## Figures and Tables

**Table 1:** Baseline patient characteristics and comorbidities of the entire cohort (N = 794) and the three groups defined according to the level of ER expression.

Parameter	Entire Series (*n* = 794)	ER-Negative/Low (0–10%) (*n* = 103)	ER-Intermediate (11–90%) (*n* = 91)	ER-High (91–100%) (*n* = 600)	*p*
Mean age (*n* = 793)	65.15 (±12.8)	61.6 (±14.9)	59.8 (±14.9)	66.5 (±11.72)	<0.00001
Age					
>65 years old	396 (49.9%)	41 (39.8%)	33 (36.3%)	322 (53.7%)	0.0007
≤65 years old	397 (50.1%)	62 (60.2%)	58 (63.7%)	277 (46.3%)	
Menopause status (*n* = 793)					
Post-menopausal	701 (88.4%)	87 (84.5%)	69 (75.8%)	545 (90.9%)	<0.00001
Pre-menopausal	92 (11.6%)	16 (15.5%)	22 (24.2%)	54 (9.01%)	
Mode of detection (*n* = 710)					
Clinical/Self	337 (47.5%)	60 (67%)	57 (64%)	220 (41.4%)	<0.00001
Screening	373 (52.5%)	30 (33%)	32 (36%)	311 (58.6%)	
Obesity (*n* = 752)					
Obese (BMI > 30)	323 (43%)	36 (37.5%)	39 (47.6%)	248 (43.2%)	0.38
Non-obese (BMI ≤ 30)	429 (57%)	60 (62.5%)	43 (52.4%)	326 (56.8%)	
Diabetes (*n* = 784)					
Yes	106 (13.5%)	9 (8.7%)	10 (11.6%)	87 (14.6%)	0.23
No	678 (86.5%)	94 (92.3%)	76 (88.4%)	508 (85.4%)	
Hypertension (*n* = 785)					
Yes	284 (36.2%)	29 (28.2%)	24 (27.6%)	231 (38.8%)	0.02
No	501 (63.8%)	74 (71.8%)	63 (72.4%)	364 (61.2%)	
Dyslipidemia (*n* = 785)					
Yes	147 (18.7%)	15 (14.6%)	14 (16.1%)	118 (19.8%)	0.35
No	638 (81.3%)	88 (85.4%)	73 (83.9%)	477 (80.2%)	

**Table 2:** Baseline tumor clinicopathologic characteristics of the entire cohort (N = 794) and the three groups defined according to the level of ER expression. LN: Lymph node, IDC: Invasive ductal carcinoma, ILC, Invasive lobular carcinoma, LVI: Lymphovascular invasion, PNI: Perineural invasion, RS: Recurrence score. Other histologies included mucinous, metaplastic, tubular carcinomas, and adenocarcinoma not otherwise specified.

Parameter	Entire Series (*n* = 794)	ER-Negative/Low (0–10%) (*n* = 103)	ER-Intermediate (11–90%) (*n* = 91)	ER-High (91–100%) (*n* = 600)	*p*
Stage (*n* = 777)					
I	400 (51.5%)	32 (31%)	39 (44.3%)	329 (55.9%)	<0.00001
II	240 (30.9%)	35 (34%)	23 (26.1%)	182 (30.9%)	
III	50 (6.4%)	7 (6.8%)	4 (4.6%)	39 (6.6%)	
IV	87 (11.2%)	26 (25.2%)	22 (25%)	39 (6.6%)	
Tumor size (*n* = 700)					
>2 cm	279 (39.9%)	42 (71.2%)	37 (46.2%)	200 (35.6%)	<0.00001
≤2 cm	421 (60.1%)	17 (28.8%)	43 (53.8%)	361 (64.4%)	
LN status (*n* = 708)					
Positive	183 (25.8%)	22 (25%)	25 (30.9%)	136 (25.2%)	0.54
Negative	525 (74.2%)	66 (75%)	56 (69.1%)	403 (74.8%)	
Histology (*n* = 794)					
IDC	591 (74.4)	89 (86.4)	62 (68.1)	440 (73.3)	0.001
ILC/Mixed	104 (13.1)	2 (1.9)	12 (13.2)	90 (15)	
Other	99 (12.5)	12 (11.7)	17 (18.7)	70 (11.7)	
Grade (*n* = 709)					
1–2	463 (65.3%)	9 (10.6%)	27 (37.5%)	427 (77.4%)	<0.00001
3	246 (34.7%)	76 (89.4%)	45 (62.5%)	125 (22.6%)	
LVI (*n* = 722)					
Present	49 (6.8%)	7 (7.7%)	11 (14.3%)	31 (5.6%)	0.01
Absent	673 (93.2%)	84 (92.3%)	66 (85.7%)	523 (94.4%)	
PNI (*n* = 725)					
Present	12 (1.7%)	0 (0.00%)	3 (3.9%)	9 (1.6%)	0.27
Absent	713 (98.3%)	91 (100%)	74 (96.1%)	548 (98.4%)	
ER histoscore (*n* = 794)					
>240	606 (76.3%)	0 (0%)	20 (22%)	586 (97.7%)	<0.00001
≤240	188 (23.7%)	103 (100%)	71 (78%)	14 (2.3%)	
PR histoscore (*n* = 751)					
>60	440 (58.6%)	0 (0%)	30 (33.3%)	410 (73.5%)	<0.00001
≤60	311 (41.4%)	103 (100%)	60 (66.7%)	148 (26.5%)	
HER2 status (*n* = 794)					
Positive	97 (12.2%)	25 (24.3%)	33 (36.3%)	39 (6.5%)	<0.00001
Negative	697 (87.8%)	78 (75.7%)	58 (63.7%)	561 (93.5%)	
Oncotype Dx RS (*n* = 284)					
≤18	203 (71.5%)	0	8 (38.1%)	195 (75.3%)	<0.00001
19–24	42 (14.8%)	0	2 (9.5%)	40 (15.4%)	
>24	39 (13.7%)	4 (100%)	11 (52.4%)	24 (9.3%)	

**Table 3:** Therapies used in the patients in the entire series and in groups according to the levels of ER expression.

Parameter	Entire Series	ER-Negative/Low (0–10%)	ER-Intermediate (11–90%)	ER-High (91–100%)	*p*
Surgery type (*n* = 729)					
Lumpectomy	513 (70%)	42 (46.7)	49 (62%)	422 (75.4%)	<0.00001
Mastectomy	216 (30%)	48 (53.3%)	30 (38%)	138 (24.6%)	
Adjuvant hormonal therapy (*n* = 790)					
Yes	617 (78.1%)	13 (12.6%)	70 (80.5%)	534 (89%)	<0.00001
No	173 (21.9%)	90 (87.4%)	17 (19.5%)	66 (11%)	
Adjuvant radiotherapy (*n* = 790)					
Yes	548 (69.4%)	61 (59.2%)	56 (64.37%)	431 (71.8%)	0.02
No	242 (30.6%)	42 (40.8%)	31 (35.63%)	169 (28.2%)	
Adjuvant chemotherapy (*n* = 790)					
Yes	269 (34.1)	76 (73.8%)	45 (52%)	148 (24.7%)	<0.00001
No	521 (65.9%)	27 (26.2%)	42 (48%)	452 (75.3%)	

**Table 4:** Survival outcomes of the patients in the entire series and in groups according to the level of ER expression. IQR: interquartile range.

Parameter	Entire Series	ER-Negative/Low (0–10%)	ER-Intermediate (11–90%)	ER-High (91–100%)	*p*
Relapse rate *n* = 739	87 (11.8%)	26 (28.9%)	13 (16.5%)	48 (8.4%)	<0.00001
Mortality rate *n* = 794	118 (14.9%)	30 (29.1%)	21 (23.1%)	67 (11.7%)	<0.00001
Median time to relapse (months, IQR) *n* = 739	46 (26–74)	38 (21.2–61)	32 (22–65.5)	49 (30–75)	0.001
Median survival time (months, IQR) *n* = 794	47(27–74.5)	41 (21.5–64)	37 (18.5–67.5)	49 (30–76)	0.002

**Table 5:** Multivariate Cox proportional hazards survival regression analysis for OS (*n* = 675). ER and PR variables were entered as a percentage of positive tumor cells. CI: confidence interval.

Variable	Coefficient	Hazard Ratio	95% CI	*p*
Age	0.046	1.04	1.02–1.06	0.0001
ER	−0.001	0.99	0.98–1.00	0.76
PR	−0.001	0.99	0.99–1.00	0.78
Stage	0.597	1.81	1.33–2.47	0.0001
Grade	0.592	1.80	1.14–2.86	0.01
Histology	0.375	1.45	0.98–2.16	0.06
Hormonal treatment	−0.906	0.40	0.19–0.84	0.01

**Table 6:** Multivariate Cox proportional hazards survival regression analysis for RFS (*n* = 666). ER and PR variables were entered as a percentage of positive tumor cells. CI: confidence interval.

Variable	Coefficient	Hazard Ratio	95% CI	*p*
Age	0.0005	1.00	0.98–1.01	0.95
ER	−0.011	0.98	0.97–0.99	0.03
PR	0.003	0.99	0.98–1.00	0.4
Stage	0.704	2.02	1.46–2.79	0.00001
Grade	0.279	1.32	0.84–2.07	0.22
Histology	0.357	1.42	0.97–2.09	0.06
Hormonal treatment	−0.561	0.57	0.26–1.23	0.15

## Data Availability

Data are contained within the article.

## Supplementary Materials

The following supporting information can be downloaded at: https://www.mdpi.com/article/10.3390/cancers17132252/s1, Table S1: Baseline patient characteristics of the cohort with intermediate ER expression (11–90%, n = 91) and the three sub-cohorts with ER level of expression between 11% and 30% (intermediate/low group), 31% and 60% (intermediate/middle group), and 61% to 90% (intermediate/high group); Table S2: Baseline tumor clinicopathologic characteristics of the cohort with intermediate ER expression (11–90%, n = 91) and the three sub-cohorts with ER level of expression between 11% and 30% (intermediate/low group), 31% and 60% (intermediate/middle group), and 61% to 90% (intermediate/high group); Table S3: Multivariate Cox proportional hazards survival regression analysis for OS (n = 705) with stage, age, grade and ER level of expression as variables; Table S4: Multivariate Cox proportional hazards survival regression analysis for RFS (n = 694) with stage, age, grade and ER level of expression as variables.
