# Supplementary material for: Clinical and Pathological Features and Survival Outcomes of Breast Cancers with Intermediate ER Expression
Source: Cancers (Basel). 2025 Jul 5;17(13):2252. doi: 10.3390/cancers17132252 (PMC12248481; doi:10.3390/cancers17132252)
Supplement: Supplementary file 1 [file cancers-17-02252-s001.zip › cancers-3685834-supplementary.pdf]

cancers

MDPI

Table S1. Baseline patient characteristics of the cohort with intermediate ER expression (11-90%, n = 91) and the three sub-cohorts with ER level of expression between 11% and 30% (intermediate/ low group), 31% and 60% (intermediate/ middle group), and 61% to 90% (intermediate/ high group).

Parameter	ER intermediate (11-90%) (n=91)	ER intermediate low (11-30%) (n=19)	ER intermediate middle (31-60%) (n=11)	ER intermediate high (61-90%) (n=61)	P
Mean age (n=91)	59.8 (±14.9)	62.7 (±14.9)	62.9 (±14.5)	57.7 (±15.7)	0.34
Age					
>65 years old	33 (63.7%)	8 (42.1%)	3 (27.3%)	22 (36.1%)	0.72
≤65years old	58 (36.3%)	11 (57.9%)	8 (72.7%)	39 (63.9%)	
Menopause status (n=91)					
Post-menopausal	69 (75.8%)	16 (84.2%)	9 (81.8%)	44 (72.1%)	0.5
Pre-menopausal	22 (24.2%)	3 (15.8%)	2 (18.2%)	17 (27.9%)	
Mode of detection (n=89)					
Clinical/ Self	57 (64.0%)	16 (84.2%)	5 (50.0%)	36 (60.0%)	0.27
Screening	32 (36.0%)	3 (15.8%)	5 (50.0%)	24 (40.0%)	
Obesity (n= 82)					
Obese (BMI>30)	39 (47.6%)	4 (23.5%)	5 (55.6%)	30 (53.6%)	0.03
Non-obese (BMI≤ 30)	43 (52.4%)	13 (76.5%)	4 (44.4%)	26 (46.4%)	

Table S2. Baseline tumor clinicopathologic characteristics of the cohort with intermediate ER expression (11-90%, n = 91) and the three sub-cohorts with ER level of expression between 11% and 30% (intermediate/ low group), 31% and 60% (intermediate/ middle group), and 61% to 90% (intermediate/ high group). .

Parameter	ER intermediate (11-90%) (n=91)	ER intermediate low (11-30%) (n=19)	ER intermediate middle (31-60%) (n=11)	ER intermediate high (61-90%) (n=61)	p
Stage (n = 88)					
I	39 (44.3%)	7 (38.9%)	6 (60.0%)	26 (43.3%)	0.80
II	23 (26.1%)	5 (27.8%)	1 (10.0%)	17 (28.4%)	
III	4 (4.5%)	0 (0%)	1 (10.0%)	3 (5.0%)	
IV	22 (25.1%)	6 (33.3%)	2 (20.0%)	14 (23.3%)	
Tumor size (n=80)					
>2 cm	37 (46.2%)	8 (50%)	3 (27.3%)	26 (49.1%)	0.39

 https://doi.org/10.3390/cancers17132252

≤2cm	43 (53.8%)	8 (50%)	8 (72.7%)	27 (50.9%)	
LN status (n = 81)					
Positive	25 (30.9%)	7 (43.8%)	2 (20%)	16 (29.1%)	0.35
Negative	56 (69.1%)	9 (56.2%)	8 (80%)	39 (70.9%)	
Histology (n=91)					
IDC	62 (68.1%)	15 (78.9)	6 (54.5)	41 (67.2)	0.61
ILC/Mixed	12 (13.2%)	1 (5.3)	2 (18.2)	9 (14.8)	
Other	17 (18.7%)	3 (15.8)	3 (27.3)	11 (18.0)	
Grade (n = 72)					
1-2	27 (37.5%)	4 (28.6%)	5 (71.4%)	18 (35.3%)	0.13
3	45 (62.5%)	10 (71.4%)	2 (28.6%)	33 (64.7%)	
LVI (n = 77)					
Present	11 (14.3%)	4 (23.5%)	0 (0.0%)	7 (14.0%)	0.52
Absent	66 (85.7%)	13 (76.5%)	10 (100.0%)	43 (86.0%)	
PNI (n = 77)					
Present	3 (3.9%)	1 (5.9%)	0 (0.0%)	2 (4.0%)	0.77
Absent	74 (96.1%)	16 (94.1%)	10 (100.0%)	48 (96.0%)	
ER histoscore (n = 91)					
>240	20 (22.0%)	0 (0.0%)	0 (0.0%)	20 (32.8%)	0.01
≤240	71 (78.0%)	19 (100.0%)	11 (100.0%)	41 (67.2%)	
PR histoscore (n=90)					
>60	30 (33.3%)	0 (0.0%)	1 (9.1%)	29 (48.3%)	0.0003
≤60	60 (66.7%)	19 (100.0%)	10 (90.9%)	31 (51.7%)	
HER2 status (n = 91)					
Positive	33 (36.3%)	12 (63.2%)	3 (27.3%)	18 (29.5%)	0.02
Negative	58 (63.7%)	7 (36.8%)	8 (72.7%)	43 (70.5%)	
Oncotype Dx RS (n=21)					
≤18	8 (38.1%)	0 (0%)	1 (25%)	7 (43.8%)	0.85
19-24	2 (9.5%)	0 (0%)	0 (0%)	2 (12.5%)	
>24	11 (52.4%)	1 (100%)	3 (75%)	7 (43.7%)	

Table S3. Multivariate Cox proportional hazards survival regression analysis for OS (n= 705) with stage, age, grade and ER level of expression as variables. ER variable was entered as percentage of positive tumor cells. CI: Confidence interval.

Variable	Coefficient	Hazard Ratio	95% CI	p
Stage	0.6026	1.82	1.34-2.48	0.0001
Age	0.0451	1.04	1.02-1.06	0.00001
Grade	0.5791	1.78	1.09-2.9	0.02
ER	-0.0081	0.99	0.98-0.99	0.01

Table S4. Multivariate Cox proportional hazards survival regression analysis for RFS (n= 694) with stage, age, grade and ER level of expression as variables. ER variable was entered as percentage of positive tumor cells .CI: Confidence interval.

Variable	Coefficient	Hazard Ratio	95% CI	p
Stage	0.7465	2.10	1.51-2.93	0.00001
Age	-0.0003	0.99	0.98-1.01	0.97
Grade	0.2315	1.26	0.80-1.96	0.3
ER	-0.0351	0.98	0.98-0.99	0.00001
